# ZnO Porous Nanosheets with Partial Surface Modification for Enhanced Charges Separation and High Photocatalytic Activity Under Solar Irradiation

**DOI:** 10.1186/s11671-019-2981-3

**Published:** 2019-05-02

**Authors:** Yanhua Tong, Shilian Lai, Fan Wu, Yuhua Guo, Haifeng Chen, Guoxiang Pan, Jingwei Sun

**Affiliations:** 1 Department of Materials and Chemistry, Huzhou University, No.759 East 2nd Road, Huzhou, 313000 China; 2 School of Science and Key Lab of Optoelectronic Materials and Devices, Huzhou University, No.759 East 2nd Road, Huzhou, 313000 China

**Keywords:** ZnO, Nanosheets, Surface-modifying, Photocatalyst, KE-7B

## Abstract

ZnO porous nanosheets (PNSs) with partial surface modification were fabricated by means of depositing amorphous BiVO_4_ on basic zinc carbonate nanosheets followed by calcining at 500 °C. At low levels of anchored amorphous BiVO_4_, the surface of ZnO PNSs was partially evolved into Bi_3.9_Zn_0.4_V_1.7_O_10.5_ (BZVO). The measurements for photocurrent and photoluminescence demonstrate that partial-surface BZVO-modified ZnO PNSs (ZB_0.01) could significantly inhibit the recombination of photoinduced carriers. This should be ascribable to the driving from surface potential difference produced by non-junction part and vertical p-n BZVO/ZnO junction part on the surface of ZB_0.01. Furthermore, the photocatalytic efficiency in degradation of reactive brilliant red for ZB_0.01 under weak solar irradiation is about 8 times higher than that under strong visible-light illumination. The discussion regarding reasons for this enhancement demonstrates that each component in photocatalysts having rational valence-band maximum and conduction-band minimum energy levels is essential to obtain high-activity sunlight-driven catalysts.

**Electronic supplementary material:**

The online version of this article (10.1186/s11671-019-2981-3) contains supplementary material, which is available to authorized users.

## Introduction

Semiconductor photocatalysis has attracted massive research interest since its solar-energy conversion is identified as a robust new method for purification and environmental protection at lower cost and energy consumption [1]. There are two central issues in terms of this research: one is committed to the extension of light absorption of photocatalysts to the visible-light region, so as to maximize the utilization of the solar spectrum; another is restricting the recombination of photoinduced electron-hole pairs to enhance quantum efficiency. As is known, coupling a wide band-gap semiconductor with a visible-light-responsive semiconductor, having a rational band alignment, is making headlines in synchronously solving the two above-mentioned issues. In the recent 10 years, many visible-light-driven composite photocatalysts have been successfully developed, such as TiO_2_-based CdS/In_2_O_3_/Ag_3_PO_4_ [2–4], ZnO-based CdS/NiO/γ-Fe_2_O_3_/Cu_2_O/BiVO_4_ [5–10], WO_3_-based CuO [11], SnO_2_/Sn_2_Ta_2_O_7_ [12], and bismuth or silver-based heterojunction nanostructures. Under visible-light illumination, all these heterojunction nanostructures showed enhanced photocatalytic activity in comparison with their single-component counterparts. However, an internal electric field can be formed only if the size of composite photocatalysts is two times larger than the width of the space-charge region (> 100 nm) [13], which has to lower the specific surface of photocatalysts. Besides, it is hard to build a perfect contact interface for making heterojunctions. These would give rise to limited activity in the photocatalytic process.

Recently, the appeal of ultrathin two-dimensional (2D) materials as an emergent class of nanomaterial stems from the huge surface area, a large fraction of low-coordinated surface atoms and ultrathin thickness for potential photocatalytic application [14, 15]. Many efforts have corroborated that ultrathin 2D or even monolayered nanosheets exhibit good photocatalytic activity in the degradation of organic pollutants [16–19], hydrogen production [20–23], and CO_2_ reduction [24–26]. Surface engineering for 2D ultrathin nanosheets, including doping [16, 20], loading carbon quantum dots [18, 22], and chemical modification [23, 25, 26], not only enhances the separation of photogenerated carriers but also holds 2D nanomaterials features. As it should be, surface-engineered 2D nanomaterials perform extraordinary photocatalytic activity and good stability due to no existing mutational interface. Yet, there were scarce investigations regarding surface-engineered 2D ZnO nanomaterials for photocatalytic applications.

ZnO as a typical traditional photocatalyst has been studied because of high photosensitivity, low cost, and environmental friendliness. In order to improve its visible-light photocatalytic activity, many attempts mainly concentrated on constructing heterojunctions [5–10] and element doping (C, S, Al, Mg [27–30], etc.). However, these studies were just based on ZnO zero-dimensional and three-dimensional nanostructures. Also, good sunlight-driven photocatalytic properties for these ZnO-based photocatalysts have not been obtained by now. In this case, it matters to employ a method to fabricate 2D ZnO nanostructures with surface engineering to substantially enhance photocatalytic behaviors of ZnO irradiated by sunlight.

Here, we demonstrated a new method for constructing junction and non-junction parts on the surface of ZnO porous nanosheets (PNSs) with controlled content, which exhibited high photocatalytic activity under solar irradiation. We followed a strategy of anchoring amorphous BiVO_4_ on 2D Zn_5_(CO_3_)_2_(OH)_6_ nanosheets and subsequent conversion to Bi_3.9_Zn_0.4_V_1.7_O_10.5_ (BZVO) domains embedding in the surface of ZnO PNSs by heat treatment. The control over size and distribution of BZVO domains was accomplished by a controlled amount of anchored BiVO_4_. At low levels of BZVO, the surface of ZnO PNSs exists a potential difference among junction and non-junction parts being a benefit to push the separation of photoinduced carriers, leading to enhanced photocatalytic activity under either visible-light or solar irradiation. It makes sense that partial-surface BZVO-modified ZnO PNSs display remarkably enhanced photocatalytic degradation of reactive brilliant red under weak solar irradiation with respect to that under strong visible light illumination. The reasons for this enhancement were discussed in detail.

## Methods

All chemicals of analytical grade were purchased from Aladdin Reagent Co. Ltd., Shanghai, China, and used as received without further purification. Distilled water has been used for synthesis and photocatalytic measurement.

### Synthesis of BiVO_4_-modifying ZnO PNSs

A strategy for the preparation of BiVO_4_-modifying ZnO PNSs was the deposition of BiVO_4_ on the surface of Zn_5_(CO_3_)_2_(OH)_6_ (ZCH) nanosheets followed by calcining. The synthesis of ZCH nanosheets was according to reference [31]. A whole portion of as-prepared ZCH nanosheets was well dispersed in 100 mL distilled water under vigorous stirring and then a measured amount of (NH_4_)_3_VO_4_ was dissolved in this mixture. Part of VO_4_^3−^ ions would be adsorbed on the surface of ZCH nanosheets under electrostatic interaction. Next, a certain amount of NaHCO_3_ was added to maintain the pH value of the mixture in the range of 6–7. Subsequently, required amount of Bi(NO_3_)_3_·5H_2_O dissolved in ethylene glycol was dropwise added to the above-made mixture, the amount of which was varied to get serial samples with varying molar ratios of Bi to Zn (0.005:1, 0.01:1, 0.02:1, 0.05:1, 0.1:1, 0.2:1). The resulting ZCH-BiVO_4_ complexes were centrifuged, washed thoroughly with distilled water, and then dried at 65 °C for 12 h.

BiVO_4_-modifying ZnO PNSs with different Bi/Zn molar ratios were obtained after a series of ZCH-BiVO_4_ combined samples were calcined at 500 °C for 2 h. The as-prepared surface-modified ZnO PNSs with the Bi/Zn molar ratio of 0.005:1, 0.01:1, 0.02:1, 0.05:1, 0.1:1, and 0.2:1 were denoted as ZB_0.005, ZB_0.01, ZB_0.02, ZB_0.05, ZB_0.1, and ZB_0.2, respectively. The color of these as-obtained samples was gradually deepened depending on the increasing Bi content. For comparison, pristine BiVO_4_ was prepared by the same process without ZCH nanosheets.

### Sample Characterization and Measurements

Powder X-ray diffraction (XRD) patterns for different samples were collected at a scan rate of 0.02 2*θ* s^−1^ ranging 5~80°, using an XD-6 diffractometer with a conventional X-ray tube (Cu Kα 36 kV, 20 mA) in transmission mode. Transmission electron microscopy (TEM), high-resolution TEM (HRTEM), high-angle annular dark-field scanning TEM (HAADF-STEM), and the corresponding energy dispersive spectroscopy (EDS) mapping analysis were performed on a JEOL JEM-ARM200F TEM/STEM with a spherical aberration corrector. X-ray photoelectron spectroscopy (XPS) measurements were carried out on a Thermo Fisher Scientific Escalab 250Xi spectrometer with monochromatized Al K alpha excitation (150 W, 500 μm), and the C 1s peak at a binding energy 284.6 eV was taken as an internal standard. UV-vis diffuse reflectance spectra (DRS) were measured using a UV-vis spectrophotometer (EVOLUTION 220) with an integrating sphere under ambient conditions. Solid photoluminescence (PL) spectra were acquired on a spectrofluorometer (fluoroSENS-9000) equipped with a filter (*λ* < 360 nm) at an export of the excitation channel and another filter (*λ* > 380 nm) at an entry of the emission channel. Photocurrent measurements were recorded with a CHI 660C electrochemical workstation (CH Instruments, Inc., Shanghai) equipped with a Pt net as a counter electrode, a saturated calomel (SCE) as reference electrode and commercial indium tin oxide (ITO) as the working electrode. The measured samples were deposited on the surface of ITO with the loading mass of 2 mg/cm^2^. The photocurrent of working electrode illuminated by visible light was measured in a mixed phosphate solution at pH = 6.86 with an applied potential of 0.1 V (vs. SCE).

### Photocatalytic Evaluation

The photocatalytic activities of as-prepared samples were tested under solar irradiation, using reactive brilliant red (KE-7B) as photodegradation probe. KE-7B solution (30 mg/L, 80 mL) was placed into a 100-mL beaker followed by an addition of 16 mg of prepared sample to maintain a catalyst concentration of 0.2 g/L. This mixed solution was stirred in the dark for 30 min until the equilibrium adsorption over photocatalyst was attained. Afterwards, the whole setup was exposed to outdoor sunlight with a perfectly transparent glass plate to cover the top. The irradiation period of time is in the range from 11:00 a.m. to 2:00 p.m. during the months of July to October. Each solution of 5 ml was taken out from the system at regular intervals and centrifuged to remove the solid sample. The absorption spectra of KE-7B solutions illuminated for different time-span were tested using a UV-vis spectrophotometer (Shimadzu, 1700 UV-vis). The KE-7B content in the irradiated solution was determined by the Lambert-Beer law. The photocatalytic measurements for different photocatalysts were performed in independent experiments. The recycle adsorption and photodegradation of ZB_0.01 were evaluated by repeating experiments under the abovementioned conditions. After each run, photocatalysts were collected after washing with deionized water and absolute ethanol several times, separately, in order to remove the adsorbed degradation products.

In comparison with sunlight, visible-light photocatalytic activity was measured by similar procedures. KE-7B solution (10 mg/L, 50 mL) and photocatalyst (10 mg) were used. The visible light was produced from a 300-W Xenon lamp (Model CEL-HXF300) with a UVIRCUT 420 filter.

### Examination of reactive species

Certain amounts of scavengers were introduced into the KE-7B solution prior to adding photocatalyst and the following procedures were the same as those in photodegradation.

## Results and Discussion

**Fig. 1:**
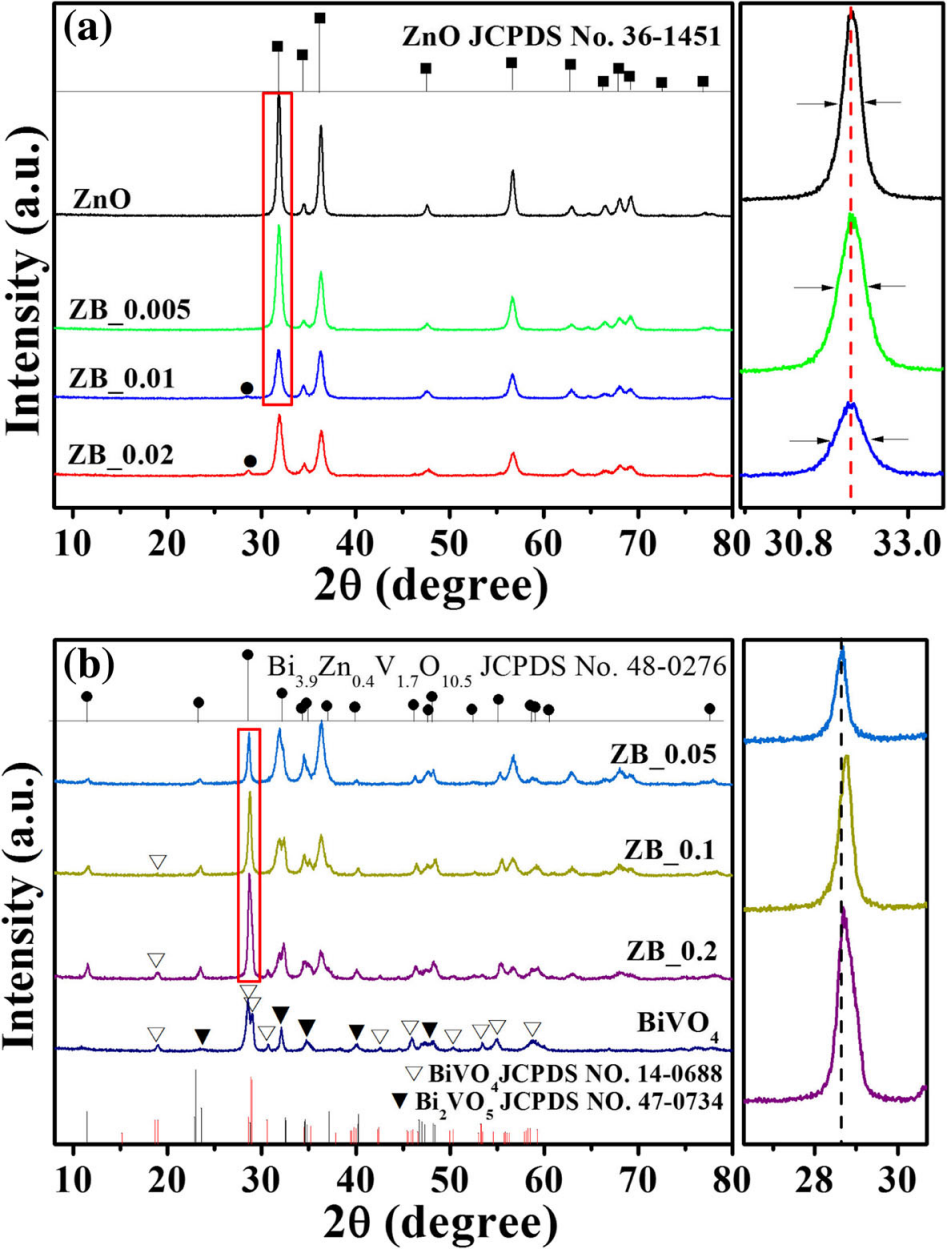
XRD patterns of ZnO, BiVO_4,_ and BiVO_4_-modifying ZnO with different Bi/Zn molar ratios. The enlarged diffraction peaks at 31.8° of ZnO (100) for ZnO, ZB_0.005, and ZB_0.01, and the amplified diffraction peaks at 28.6° of BZVO solution for ZB_0.05, ZB_0.1, and ZB_0.2 are separately shown in **a** and **b** on the right, respectively

The phase structure and purity of as-prepared products were analyzed using the powder XRD technique. Figure 1 illustrates XRD patterns of ZnO, BiVO_4_ and a series of BiVO_4_-modifying ZnO. All diffraction peaks in Fig. 1 (ZnO) exactly match well with XRD data of JCPDS card No.36-1451 for ZnO wurtzite hexagonal phase. Figure 1 (BiVO_4_) shows the diffraction peaks of resulting BiVO_4_, which can be indexed for the monoclinic phase of BiVO_4_ (JCPDS card No. 14-0688) and orthorhombic structure of Bi_2_VO_5_ (JCPDS card No. 47-0734). The diffraction intensity of the monoclinic phase is far stronger than that of the orthorhombic phase, indicating that our as-prepared BiVO_4_ is overwhelmingly composed of monoclinic phase. Figure 1 (ZB_0.005)–(ZB_0.2) show XRD patterns of stepwise formation of BZVO-modified ZnO. As the Bi/Zn molar ratio increases, the intensity of 31.8° (100) peak of ZnO is found to be gradually decreased and synchronously a new peak at 28.6° appears and is gradually intensified. This peak at 28.6° attached with other new peaks could be well assigned to tetragonal BZVO (JCPDS No. 48-0276). This suggests that a new phase of BZVO produces and grows up with the increasing Bi/Zn molar ratio. The enlarged diffraction peaks of (100) for ZB_0.005, ZB_0.01, and ZB_0.02 are separately shown in Fig. 1a on the right. It presents that increasing the content of Bi, the intensity of ZnO (100) diffraction peak is weakened and its width at half height is broadened, revealing a decrease in size for ZnO. It is because ZCH nanosheets reacting with anchored BiVO_4_ produces BZVO, sacrificing part of the ZnO phase. Upon the Bi/Zn molar ratio being > 0.05:1, apart from ZnO and BZVO phases, BiVO_4_ begins to form, as denoted by triangle symbols in Fig. 1b. The formation of BiVO_4_ can be further proved by the enlarged diffraction peak at 28.6° of BZVO (Fig. 1b on the right) which begins to broaden and be unsymmetrical upon the Bi/Zn molar ratio being higher than 0.05:1.

**Fig. 2:**
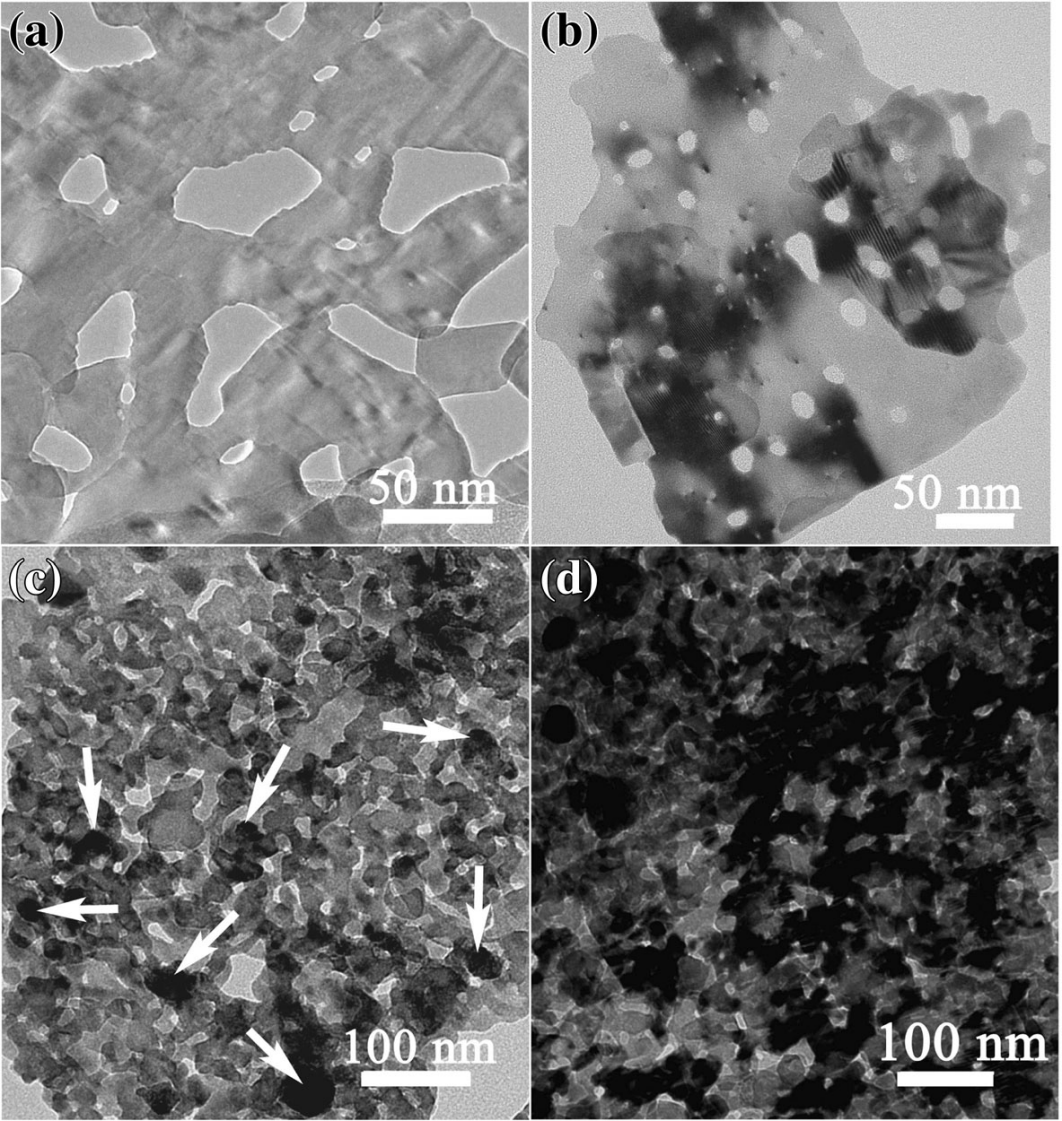
TEM images of BiVO_4_-modifying ZnO PNSs with different Bi/Zn molar ratios: **a** ZnO, **b** ZB_0.01, **c** ZB_0.05, and **d** ZB_0.2, showing the evolution of surface morphology as the increasing Bi/Zn molar ratio

The morphology of ZCH precursor was described as reference [31]. It is in the shape of sheets with near to 1 μm in width and several nanometers in thickness. After calcining at 500 °C, ZCH nanosheets evolved into ZnO nanosheets with pores that were caused by the escape of CO_2_ and H_2_O gas generated by pyrolyzing of ZCH (Fig. 2a). Shown in Fig. 2a–d are TEM images, illustrating a morphology evolution on the surface of ZnO porous nanosheets with the increasing Bi/Zn molar ratio. For pristine ZnO, the brightness in the whole porous nanosheet is almost the same and its surface is smooth (Fig. 2a). Increasing the Bi/Zn molar ratio to 0.01, remarkable brightness contrast on the same nanosheet was observed (Fig. 2b), which suggested that a new phase in the dark zone formed on the surface of the ZnO nanosheet. In combination with the results from XRD characterization, the new phase might be BZVO. Upon increasing the Bi/Zn molar ratio up to 0.05, the dark zone on the surface of porous nanosheets was enlarged and some attached particles appeared as shown by arrows (Fig. 2c). When the Bi/Zn molar ratio increased up to 0.2, much more black particles loaded on porous nanosheets were found (Fig. 2d). Because XRD characterization demonstrates that the increasing Bi/Zn molar ratio gives rise to first BZVO and then BiVO_4_ formation, it confirms that the dark zone on the surface of porous nanosheets is the BZVO phase, the black attached particles presented at high Bi/Zn molar ratio are BiVO_4_. Based on TEM and XRD results, as the initial increase of Bi/Zn ratio, only the BZVO phase grew on the surface of ZnO porous nanosheets. Upon the Bi/Zn ratio increasing up to 0.05, both BZVO phase and BiVO_4_ particles grew on ZnO porous sheets.

**Fig. 3:**
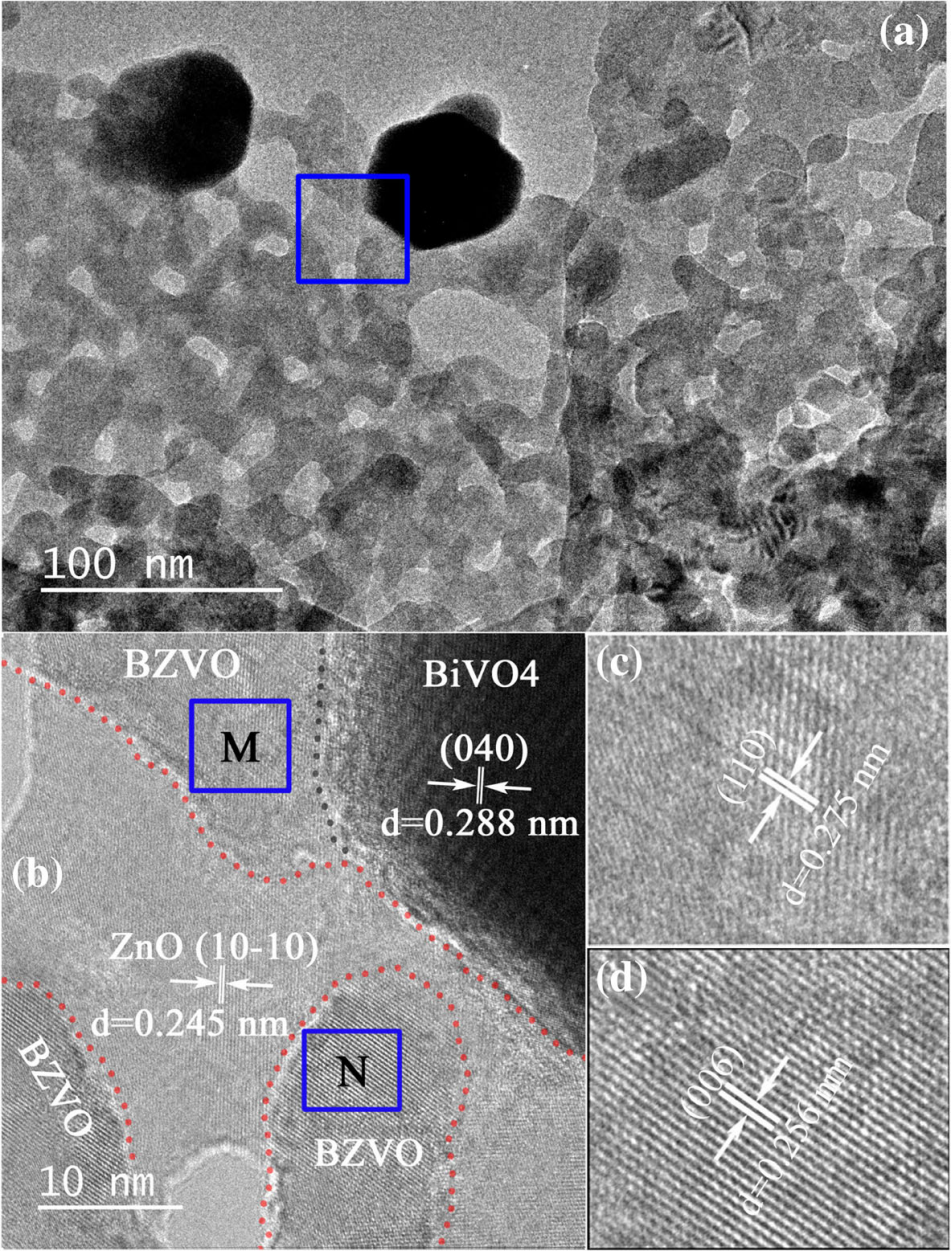
**a** TEM image of ZB_0.05, showing porous sheetlike nanostructure. **b** High-resolution TEM image for the enlarged region remarked with blue rectangle in the TEM image, clearly displaying BZVO domains embedding in ZnO lattice. Enlarged BZVO domains remarked with M and N blue rectangles are separately shown in **c** and **d**, respectively

Since the surface microstructure of ZB_0.05 contains features of both ZB_0.01 and ZB_0.2, it was typically selected for microstructural analysis. Its enlarged TEM image in Fig. 3a presents porous nanosheets loaded with few particles, consistent with the HAADF-STEM image shown in Additional file 1: Figure S1 (top). Different from the smooth surface of ZnO PNSs, that of BiVO_4_-modifying ZnO PNSs is uneven. The localized region of one porous nanosheet marked in a blue square in Fig. 3a was amplified into Fig. 3b. It illustrates three categories of domains in color including light grey, dark grey, and black. A typical lattice distance of 0.245 nm in the light-grey region can be assigned to the (10-10) facet of hexagonal ZnO. Dark-grey regions marked with blue squares containing M and N letters were correspondingly amplified in Fig. 3c and d, and their crystal fringes at 0.275-nm and 0.256-nm lattice spacing are consistent with the (110) and (006) planes of tetragonal BZVO, respectively. Fig. 3b displays that BZVO domains are embedded in ZnO lattice, in agreement with the deduction that BZVO is produced by anchored BiVO_4_ reacting with ZCH substrate. Referring to the TEM image, the black region in the HRTEM image is part of one particle. Its lattice fringes at 0.288-nm spacing match with the (040) planes of monoclinic BiVO_4_. This implies that the loaded particles on PNSs are only BiVO_4_ phase. The HRTEM characterization indicates that the surface of ZB_0.05 PNSs is composed of ZnO and BZVO domains, as well as a few loaded BiVO_4_ particles.

Considering that the Bi content in ZB_0.01 is possibly lower than the element-detecting limit of EDS and XPS, ZB_0.05 with higher Bi content was characterized by EDS and XPS. The element EDS mapping signals of Zn and O elements are much denser than that of Bi and V elements (Additional file 1: Figure S1), demonstrating that the principle component of ZB_0.05 is ZnO. The signals of Bi and V elements are almost the same and dispersed in ZnO PNSs; it also supports the conclusion from HRTEM characterization that BZVO phase is homogeneously embedded in the surface of ZnO PNSs. That only Zn mapping image doesn’t show particle-like shapes also suggests that the chemical component of particles loaded on PNSs is BiVO_4_. The results from XRD, HRTEM, and element-mapping characterization corporately demonstrate that at low levels of the Bi element, the partial surface of ZnO PNSs evolves into BZVO; at high levels of the Bi element, much more of the surface of ZnO PNSs turns into BZVO and some of which are loaded with BiVO_4_ nanoparticles.

**Fig. 4:**
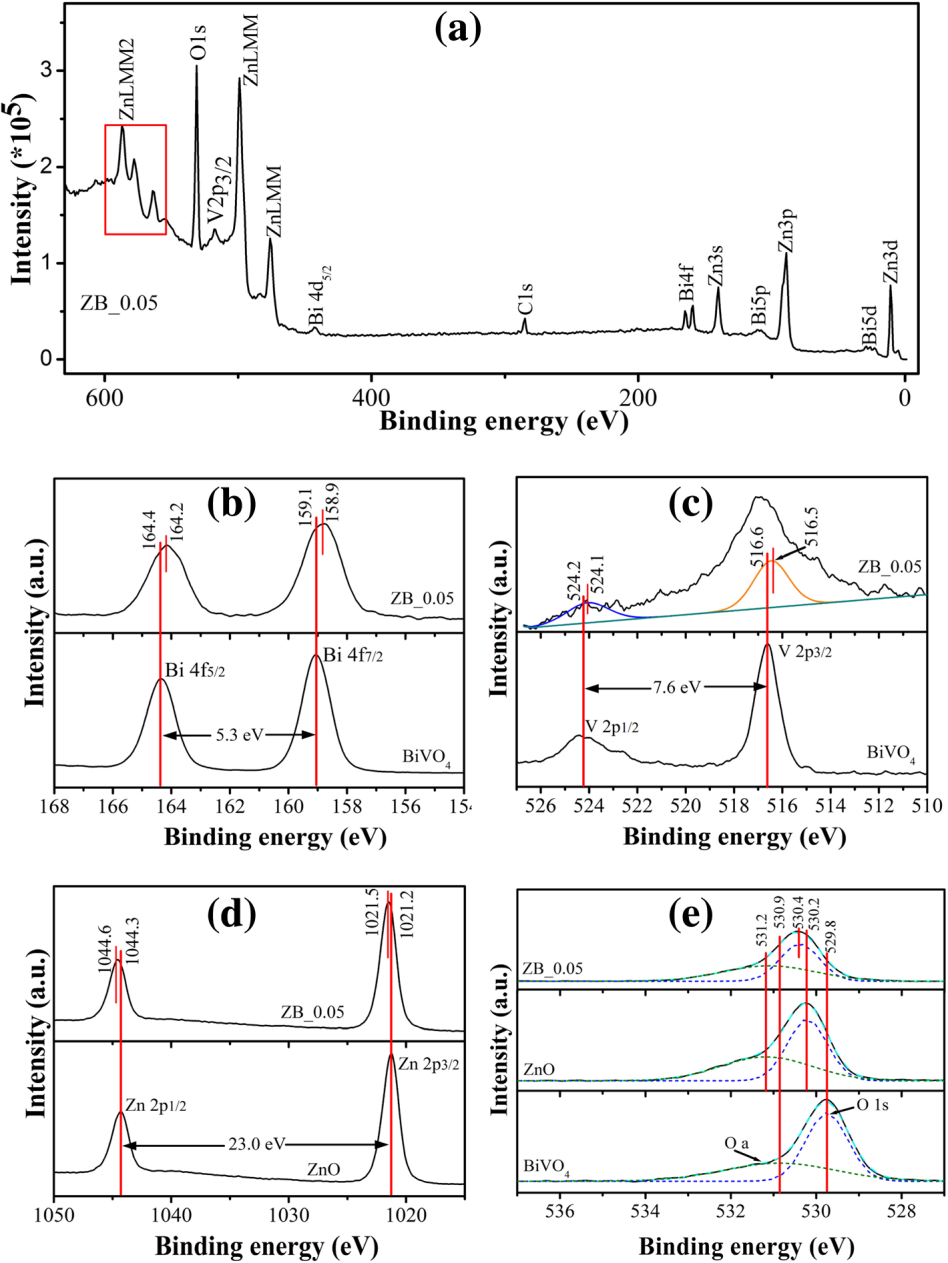
XPS whole scanning spectrum of ZB_0.05 (**a**) and corresponding fine spectra of Bi 4f (**b**), V 2p (**c**), Zn 2p (**d**), and O 1 s (**e**) of ZnO, ZB_0.05, and BiVO_4_ samples

Elements and their surface chemical states in ZB_0.05 were further analyzed by XPS. The binding energies in the presented XPS were calibrated with that of C 1s at 284.6 eV. Figure 4a presents the XPS survey spectrum of ZB_0.05, where all peaks are assigned to the elements of Zn, Bi, O, and V apart from C element from adsorbed carbon on the sample surface. For the fine spectrum of Bi 4f of ZB_0.05 in Fig. 4b, the two peaks at 164.2 eV and 158.9 eV are indexed to Bi 4f5/2 and Bi 4f7/2, respectively. The peak separation between them is 5.3 eV, indicating + 3 oxidation state of bismuth. The characteristic spin-orbit splitting of V 2p1/2 and V 2p3/2 signals was observed at 524.1 eV and 516.5 eV, respectively for ZB_0.05 (Fig. 4c). The two peak separation of ca. 7.6 eV corresponds to V^5+^ species in the sample, according to the *Handbook of X-ray Photoelectron Spectroscopy* [32]. The red shifts of Bi and V binding energies were observed from fine XPS spectra of ZB_0.05 with respect to that of pristine BiVO_4_, which are due to the fact that Zn atoms donated electrons to adjacent more electronegative Bi and V atoms and thus enhanced their core electron densities. The fine spectrum of Zn 2p for ZB_0.05 exhibits one peak at 1044.6 eV and another at 1021.5 eV, which are corresponding to Zn 2p1/2 and Zn 2p3/2, respectively (Fig. 4d). The peak separation of 23.0 eV is ascribed to the state of Zn^2+^ cations in sample. As expected, the two peaks for Zn 2p are shifted to the high binding energy by 0.3 eV in terms of that of pristine ZnO. It verifies that Zn atoms donated electrons and decreased electrons density themselves. The red shifts of Bi 4f and V 2p binding energies and the blue shift of Zn 2p binding energies can suggest that Zn atoms associating with Bi and V atoms locate in the same lattice. Shown in Fig. 4e, the high-resolution O1s spectra for ZB_0.05, ZnO and BiVO_4_ have been de-convoluted into two individual peaks: the peak at low binding energy attributed to O^2−^ type ions in lattice (denoted as O 1s), another at high binding energy associated with adsorbed O_2_ (named O a) [33, 34]. The binding energy at 530.40 eV of lattice O1s on the surface of ZB_0.05 is larger than those over pristine BiVO_4_ (529.5 eV) and ZnO (530.2 eV). It suggests that the O 1s with high binding energy has to belong to BZVO lattice for valence electrons of O element in BZVO are not only bound by V and Bi elements but also by Zn element. Accordingly, it is reasonably followed that the surface of ZB_0.05 is majorly composed of BZVO.

**Fig. 5:**
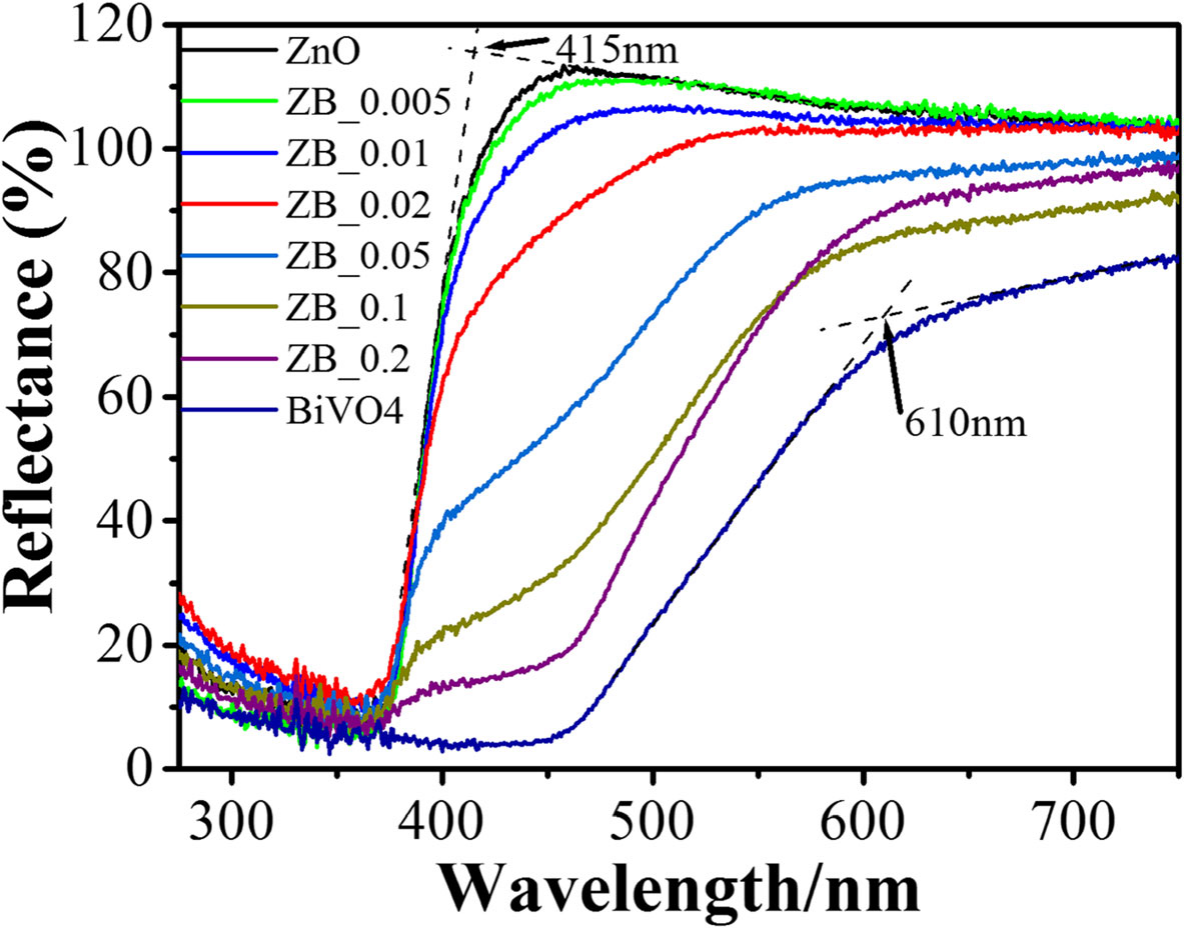
UV-vis DRS of ZnO, BiVO_4_, and BiVO_4_-modifying ZnO with different Bi/Zn molar ratios. It shows that the harvest of visible light for BiVO_4_-modifying ZnO nanosheets enhances with the increasing Bi/Zn ratio

UV-vis DRS of as-prepared samples are presented in Fig. 5. White ZnO only absorbs UV light with an abrupt sharp absorption edge at ~ 415 nm, and yellow BiVO_4_ has a strong absorption in the visible-light range of 400~500 nm, with a mild absorption edge at ~ 610 nm. With respect to ZnO, visible-light adsorption in the region of 400~550 nm for BiVO_4_-modifying ZnO is gradually harvested with the increasing Bi/Zn ratio. The density of states (DOS) for BZVO was calculated by density functional theory (see Additional file 1). Its band gap is estimated to be 2.0 eV by potential difference between valence-band maximum (VBM) and conduction-band minimum (CBM) energy levels (Additional file 1: Figure S2), which enable BZVO to absorb visible light. The harvest among UV and the visible-light region perhaps endows BiVO_4_-modifying ZnO with good solar photocatalytic activity.

**Fig. 6:**
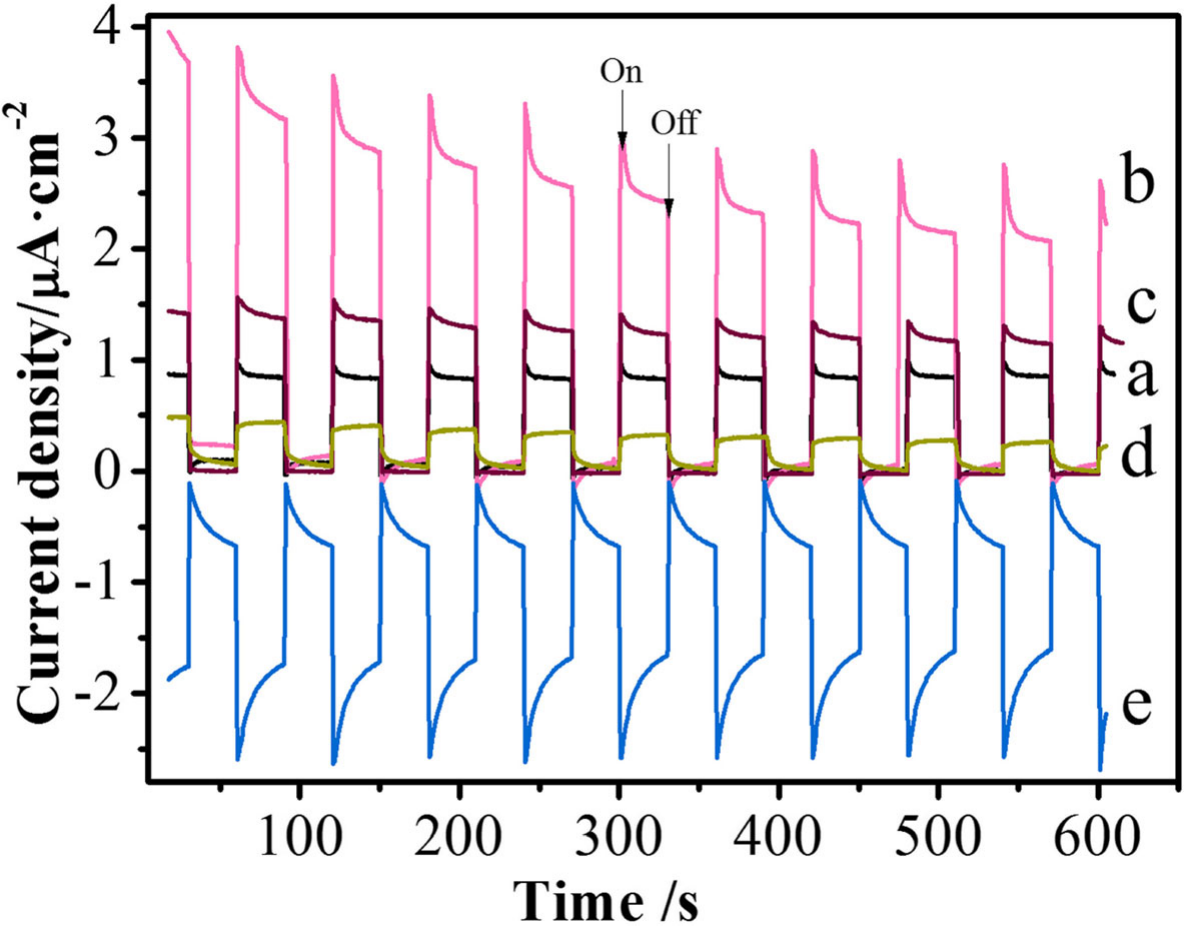
Photocurrent curves versus time: **a** ZnO, **b** ZB_0.01, **c** ZB_0.05, **d** ZB_0.2, and **e** BiVO_4_. The irradiating light was produced by the xenon lamp with a UVIRCUT 420 filter

Figure 6 shows photocurrent response curves for ZnO, BiVO_4_, ZB_0.01, ZB_0.05, and ZB_0.2 under an on/off xenon lamp attached with UVIRCUT 420 filter in ten cycles (the irradiation spectrum in Fig. 9c). It is discerned that an order for photocurrent density follows ZB_0.01 > ZB_0.05 > ZnO > ZB_0.2. This indicates that partial surface of ZnO PNSs modified by BZVO can promote to separate photoinduced carriers of ZnO PNSs, whereas a high content of BZVO solution in ZnO PNSs plays an opposite role. In order to evidence this performance of photogenerated carriers, PL spectra for these samples were measured using exciting wavelength at 330 nm (Additional file 1: Figure S3). It shows that after the surface of ZnO PNSs has been partly modified by BZVO, PL emission is by contrast weakened and even disappears for ZB_0.05 and ZB_0.01, respectively. However, at high levels of BZVO, PL emission for ZB_0.2 rises again. This also proves that partial surface modified by BZVO could be of benefit to separate photoinduced carriers of ZnO PNSs, because the attenuation of PL suggests that there is less radiative recombination and therefore the carriers could be separated.

**Fig. 7:**
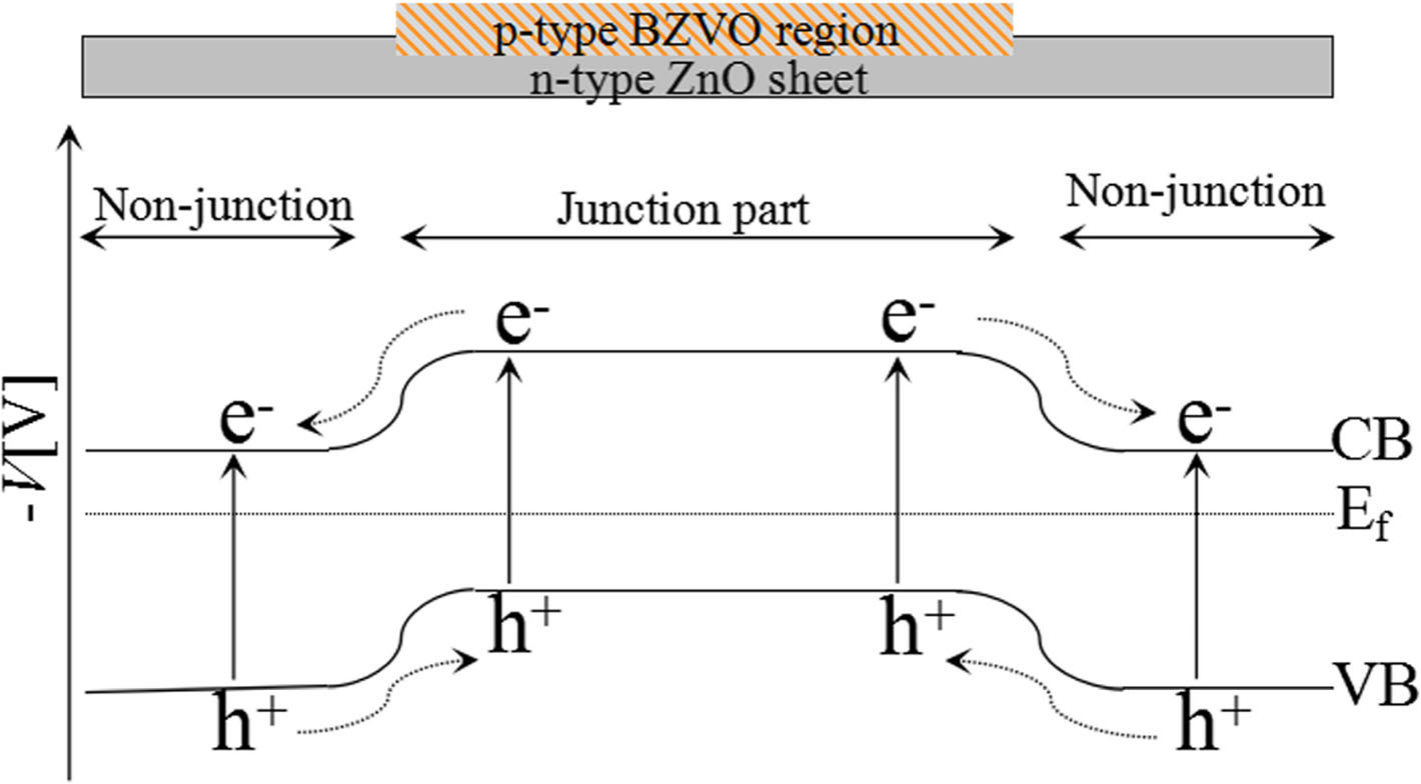
Proposed band structure for partial-surface BZVO-modified ZnO PNSs

Although ZB_0.2 with high Bi/Zn molar ratio has better harvest of visible light (Fig. 5), its photocurrent density under visible-light irradiation does not enhance. This result cannot be explained by internal electric field caused by heterogeneous structures. Figure 6 presents positive and negative photocurrent density for different samples. It is known that an n-type semiconductor produces positive photocurrent density and a p-type semiconductor produces a negative value. In this regard, the value of photocurrent density for ZnO is positive revealing that ZnO is an n-type semiconductor. On the contrary, that for BiVO_4_ is negative suggesting that it is a p-type semiconductor. Although single-phase BZVO is not successfully prepared for photocurrent measurement, a DOS calculation shows that Femi energy level of BZVO near to the valence band top (Additional file 1: Figure S2), demonstrating BZVO is also a p-type semiconductor. For low-level BiVO_4_-modifying ZnO PNSs, p-type BZVO domains embed in the lattice of n-type ZnO PNSs (Fig. 3b). This could produce longitude BZVO/ZnO p-n junction parts and ZnO non-junction parts on the surface of ZnO PNSs. Owing to space charge compensation in p-n junction parts and diffusion of carriers at the edge of non-junction parts [35], the surface potential of the BZVO/ZnO junction part is lower than that of the non-junction parts on the same ZnO sheetlike substrate (Fig. 7). These potential gradients result in specially separated reaction sites and effectively separate photoinduced electrons and holes. At high Bi/Zn ratio, the surfaces of ZnO PNSs are almost covered by BZVO (for example, ZB_0.2) and have no remarkable junction and non-junction parts to generate surface potential gradient. This is the rational reason for low-photocurrent response and high PL for ZB_0.2. The low recombination of photoinduced carriers for partly BZVO-modified ZnO PNSs is appealing for photocatalysts.

**Fig. 8:**
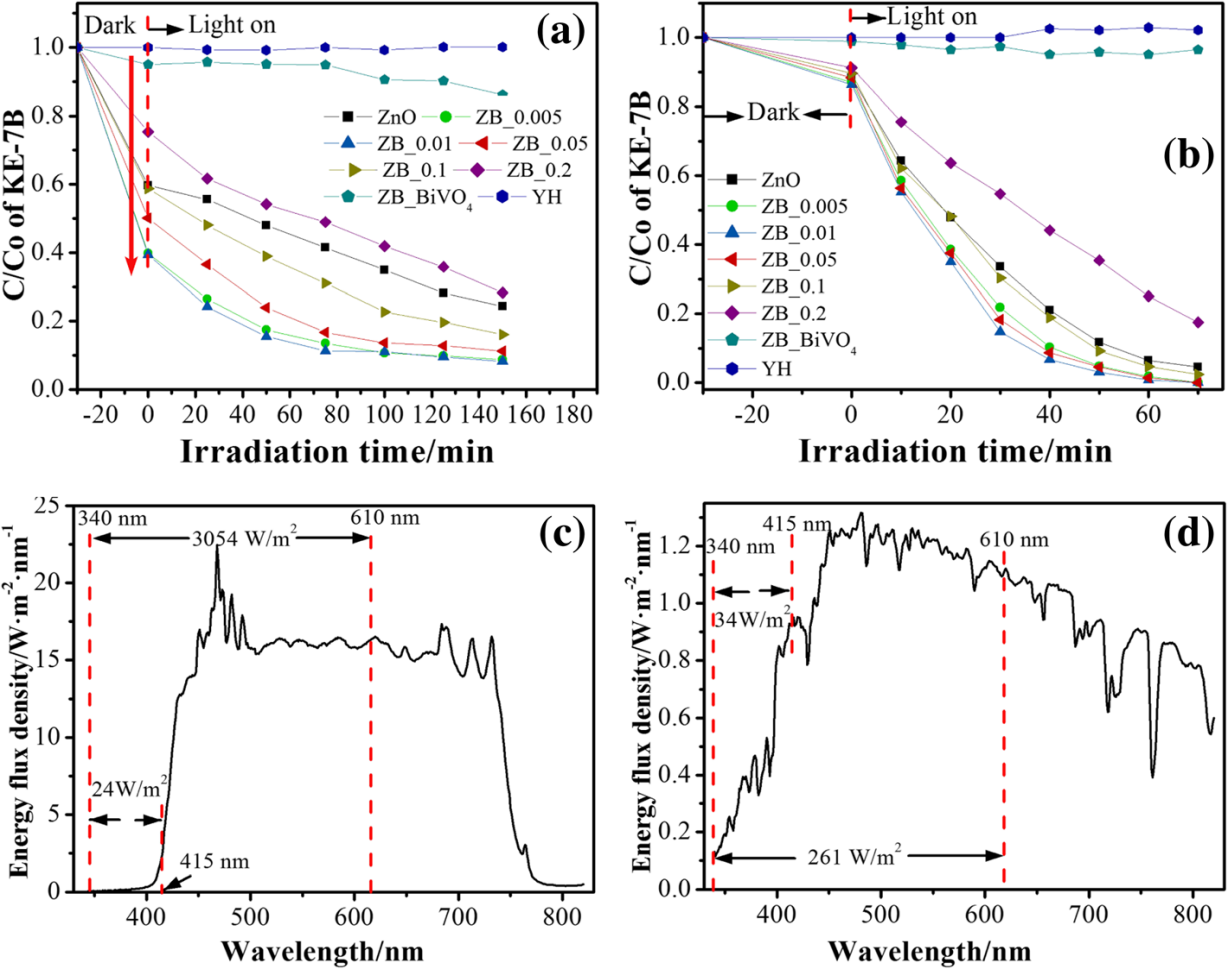
Variation of the relative content of KE-7B (C/C_0_) vs. irradiation time over ZnO, BiVO_4_, and BiVO_4_-modifying ZnO with different Bi/Zn molar ratios under visible-light (**a**) and natural sunlight (**b**) illumination. **c** Visible-light spectrum and (**d**) solar-light spectrum ranging 340~820 nm measured by a movable spectroradiometer

**Fig. 9:**
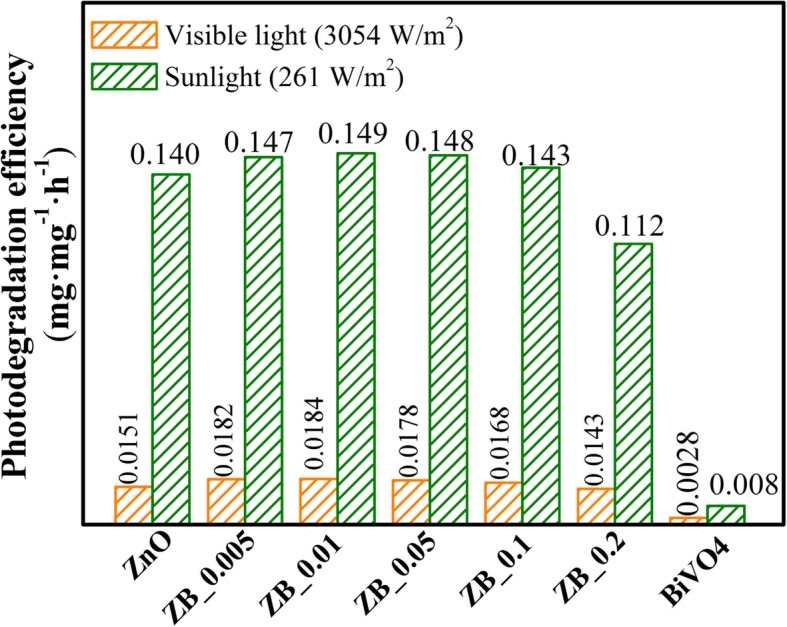
Photocatalytic degradation efficiency for ZnO, BiVO_4_, and BiVO_4_-modifying ZnO with different Bi/Zn molar ratios under visible light and natural sunlight illumination

Figure 8a and b show the adsorption and photocatalytic degradation curves of KE-7B in the presence of as-prepared samples irradiated by visible light and natural sunlight, respectively. *C*_0_ and *C* are denoted as the initial and the remaining concentrations of KE-7B aqueous solution, respectively. According to the *C*/*C*_0_ value at the irradiation time of 0 min (i.e., after the adsorption equilibrium) (Fig. 8a), the adsorption capacities (unit, mg/g) have been evaluated and follow the order as the arrow shown in Fig. 8a: ZB_0.01 (30.3) ≈ ZB_0.005 (30.1) > ZB_0.05 (25.0) > ZB_0.1(20.6) ≈ ZnO (20.2) > ZB_0.2 (12.3) > BiVO_4_ (2.5). This implies that partial-surface modification endows ZnO PNSs with better adsorption ability. In both Fig. 8a and b, blank tests show that the KE-7B concentration is almost invariable under solar or visible-light illumination, suggesting that self-decomposition has not occurred in the absence of photocatalysts. Meanwhile, pristine BiVO_4_ displays very poor photodegradation. Differently, ZnO and BiVO_4_-modifying ZnO perform photocatalytic activity under either visible light or natural solar irradiation. Photodegradation efficiency for these samples was calculated by $\left[\frac{\Delta C}{C_0}\times {W}_1\right]/\left[{W}_2\times t\right]$ formula, where *C*_0_denotes the initial concentration of KE-7B, Δ*C*refers to the variation of KE-7B concentration, *W*_1_and *W*_2_are the weight of KE-7B and photocatalyst, respectively, and *t* is irradiation time. The calculated results were illustrated in Fig. 9.

Under visible-light illumination, the degradation efficiency increases with the increasing Bi/Zn molar ratio up to 0.01 and then decreases with the ratio of > 0.01. The same degradation order was under sunlight illumination. Accordingly, the optimal Bi/Zn ratio of BiVO_4_-modifying ZnO is found to be 0.01. This should be attributed to that ZB_0.01 has the best separation of photoinduced carriers and the highest adsorption capacity of KE-7B. Compared to commercial ZnO photocatalysts with ca. 50 nm in diameter, ZB_0.01 performs more than a two-time enhancement in photodegradation efficiency, although ZB_0.01 has lower adsorption capacity than commercial ZnO (Additional file 1: Figure S5). Such a result indicates that the separation of photoinduced carriers could play a more important role in enhancing photocatalytic efficiency than adsorption capability.

More importantly, it has been noticed in Fig. 9 that catalytic efficiency under natural sunlight irradiation is significantly enhanced with respect to that under visible-light irradiation for each sample except pristine BiVO_4_. Taking ZB_0.01 as an example, solar catalytic efficiency (0.149 mg mg^−1^ h^−1^) is roughly eightfold higher than visible light (0.0184 mg mg^−1^ h^−1^). This remarkable enhancement in photocatalytic activity was analyzed by effects from irradiation spectrum and active species.

To investigate an effect of irradiation spectrum on the photocatalytic activity, the used spectra of visible light and sunlight were recorded in the range of 340~820 nm by a movable spectroradiometer (Apogee model, 110), as shown in Fig. 8c and d, respectively. The visible-light irradiation mainly ranges from 420 to 750 nm, and the sunlight radiation covers a wide range of 350~800 nm. According to the absorption edge of ZnO (415 nm), the integrated radiant flux from 340 to 415 nm that could be absorbed by ZnO is 24 and 34 W m^2^ for the used visible light and natural sunlight, respectively. According to the absorption edge of BiVO_4_ (610 nm), that from 340 to 610 nm which could be absorbed by BiVO_4_ is 3054 and 261 W m^2^ for the used visible light and natural sunlight, respectively. Unexpectedly, the strong visible-light radiant flux doesn’t enable BiVO_4_-modifying ZnO PNSs to exhibit good photocatalytic activity, but they perform excellent photocatalytic activity under weak sunlight irradiation. This result could suggest that it is UV light not visible light that plays the key role in the photocatalytic degradation of KE-7B, using BiVO_4_-modifying ZnO PNSs as photocatalysts.

To evaluate main active species in the photodegradation process, scavenger trapping experiments were carried out using ZB_0.01 as the photocatalyst under sunlight irradiation. Three types of scavengers were used to trap corresponding active species: benzoquinone (BQ) for •O_2_^−^, isopropyl alcohol (IPA) for •OH, and triethanolamine (TEOA) for h_vb_^+^. Additional file 1: Figure S4 shows the variation of KE-7B degradation in the presence of different scavengers dependent on irradiation time. In comparison with the absence of scavenger, the photocatalytic degradation efficiency reduces in the presence of BQ and TEOA, but that stays the same in the presence of IPA. This result indicates that h_vb_^+^ and •O_2_^−^ are main active species responsible for the KE-7B photodegradation over ZB_0.01 and •OH species could be ignored.

For ZnO PNSs, the value of CBM potential is − 0.49 V [31]. This value is more reducing than the formation potential of •O_2_^−^ species (O_2_ + e_cb_^−^ → •O_2_^−^_,_ − 0.33 V), which enables UV light-excited ZnO part to yield photocatalytic active •O_2_^−^ species. However, the calculated CBM potential for BZVO (0.5 V) is more oxidizing than the formation potential of •O_2_^−^ species (− 0.33 V), which cannot facilitate photogenerated electrons to be converted into active •O_2_^−^ species over BZVO region. Although the VBM potential of ZnO (2.8 V) and BZVO (2.5 V) is more oxidizing than the formation potential of •OH (h_vb_^+^ + H_2_O → •OH + H^+^_,_ 2.23 V), there is no formation of •OH species. This is because the strong adsorption of KE-7B onto the surface of BiVO_4_-modifying ZnO PNSs makes zero distance for photogenerated h_vb_^+^ species transferring to absorbed KE-7B molecules. As such, photogenerated h_vb_^+^ species would directly oxidize KE-7B molecules, not oxidizing H_2_O molecules to produce •OH species. Accordingly, photocatalytic active species for partial-surface BZVO-modified ZnO PNSs are h_vb_^+^ species produced by ZnO and BZVO parts and •O_2_^−^ species formed only by ZnO part.

Under strong visible-light irradiation, only the BZVO part on the surface of partial-surface BZVO-modified ZnO PNSs plays the major role of photocatalytic degradation because ZnO part is not responsive to visible light. Besides, photogenerated electrons could not be dissipated by O_2_ and would recombine with photoinduced holes, resulting in much less photocatalytic active species. By contrast, under solar irradiation, both BZVO and ZnO parts on BiVO_4_-modifying ZnO PNSs perform photocatalytic degradation. In addition, not only photoinduced electrons but also holes over ZnO region could turn into photocatalytic active species. As a result, partial-surface BZVO-modified ZnO PNSs perform the remarkable enhancement in catalytic efficiency under weak solar irradiation with respect to strong visible-light irradiation.

**Fig. 10:**
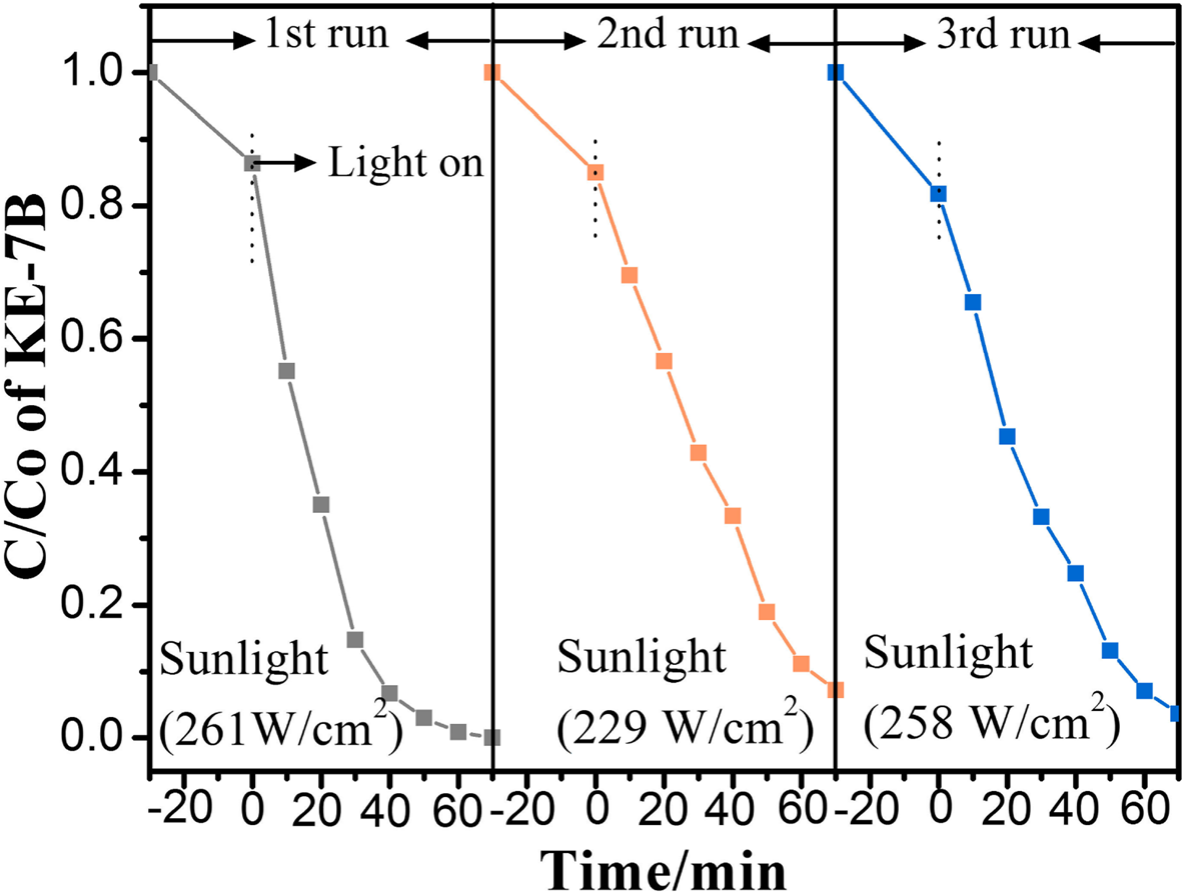
Cycling test of adsorption and sunlight-driven photocatalytic degradation for ZB_0.01, displaying tolerable photostability

The photostability of ZB_0.01 with best photocatalytic activity was separately studied, holding all other parameters invariable, except for temporary sunlight. Undergoing three successive cycles, it was found that the photodegradation percentage for ZB_0.01 still reached 96% (Fig. 10). It indicates that partial-surface BZVO-modified ZnO PNSs have tolerable stability and no serious photocorrosion during the photocatalytic oxidation process. The high sunlight-driven photocatalytic activity and stability of this ZnO PNSs with surface engineering are especially beneficial to practical application.

## Conclusions

In summary, we report an approach to obtain partial-surface BiVO_4_-modified ZnO PNSs with high photocatalytic activity under natural sunlight irradiation. Our fabrication involved anchoring amorphous BiVO_4_ phase onto ZCH nanosheets and evolving into BZVO/ZnO composite nanosheets by calcining. At intermediate BZVO content, ZB_0.01 has the best separation of photoinduced carriers, which is activated by surface potential difference from ZnO non-junction and p-n BZVO/ZnO junction parts on the surface of ZB_0.01. Apart from it, the experiment concerning the effect of irradiating light on photocatalytic activity suggests that it is essential for all single-component counterparts of composite having rational VBM and CBM levels to produce photocatalytic active species. This work not only provides a surface-modified route to separate photoinduced carriers but also can be a guide to the surface engineering of ZnO PNSs for highly desirable sunlight-driven degradation of organic pollutant.

## Additional file

Additional file 1: Supporting information. (DOC 1708 kb)

## Abbreviations

2D: Two-dimensional
BZVO: Bi_3.9_Zn_0.4_V_1.7_O_10.5_
CBM: Conduction-band minimum
DOS: Density of states
DRS: Diffuse reflectance spectra
EDS: Energy dispersive spectroscopy
HAADF-STEM: High-angle annular dark-field scanning transmission electron microscopy
HRTEM: High-resolution transmission electron microscopy
ITO: Indium tin oxide
KE-7B: Reactive brilliant red
PL: Photoluminescence
PNSs: Porous nanosheets
SCE: Saturated calomel
TEM: Transmission electron microscopy
VBM: Valence band maximum
XPS: X-ray photoelectron spectroscopy
XRD: Powder X-ray diffraction
ZCH: Zn_5_(CO_3_)_2_(OH)_6_
