# Supplementary material for: ZnO Porous Nanosheets with Partial Surface Modification for Enhanced Charges Separation and High Photocatalytic Activity Under Solar Irradiation
Source: Nanoscale Res Lett. 2019 May 2;14:151. doi: 10.1186/s11671-019-2981-3 (PMC6497717; doi:10.1186/s11671-019-2981-3)
Supplement: Supplementary file 1 — Supporting information. (DOC 1708 kb) [file 11671_2019_2981_MOESM1_ESM.doc]

**Supporting Information**

**ZnO porous nanosheets with partial surface solutionizing for enhanced charges separation and high photocatalytic activity under solar irradiation**

Yanhua Tong^1*^, Shilian Lai^1^, Fan Wu^2^, Yuhua Guo^1^, Haifeng Chen^1^, Guoxiang Pan^†^

and Jingwei Sun^1^

^1^Department of Materials and Chemistry, Huzhou University, Huzhou 313000, China

^2^School of Science and Key Lab of Optoelectronic Materials and Devices, Huzhou University, Huzhou, 313000, China

* To whom correspondence should be addressed: E-mail: [yh_tong123@126.com](mailto:yh_tong123@126.com)； Phone/Fax: +86-0572-2320685; No.759 East 2nd Road, Huzhou, Huzhou 313000, China.

**DOS by DFT calculation.**

Density of states (DOS) for BZVO was investigated based on first-principles calculations within the framework of DFT+U as implemented in the VASP code [1]. Nuclei-electron interactions were described by the projector augmented wave (PAW) pseudopotentials [2], but the electronic exchange and correlation effects were described within the generalized gradient approximation (GGA) as parameterized by Perdew, Burke, and Ernzerhof [3]. The Hubbard U corrections are used to describe 3d electrons of V element, and the U value is set to be 2.7 eV [4]. The k-point sampling of the Brillioun zone was 9×9×3 grid for Bi_3.9_Zn_0.4_V_1.7_O_10.5_ obtained by XRD measurement. The planewave basis set used had a high cut-off energy of 500 eV throughout the computations. The convergence criteria for total energy and force were 10^-5^ eV and 5×10^-2^ eV·Å^-1^, respectively. Spin polarization effect is also considered in this work.


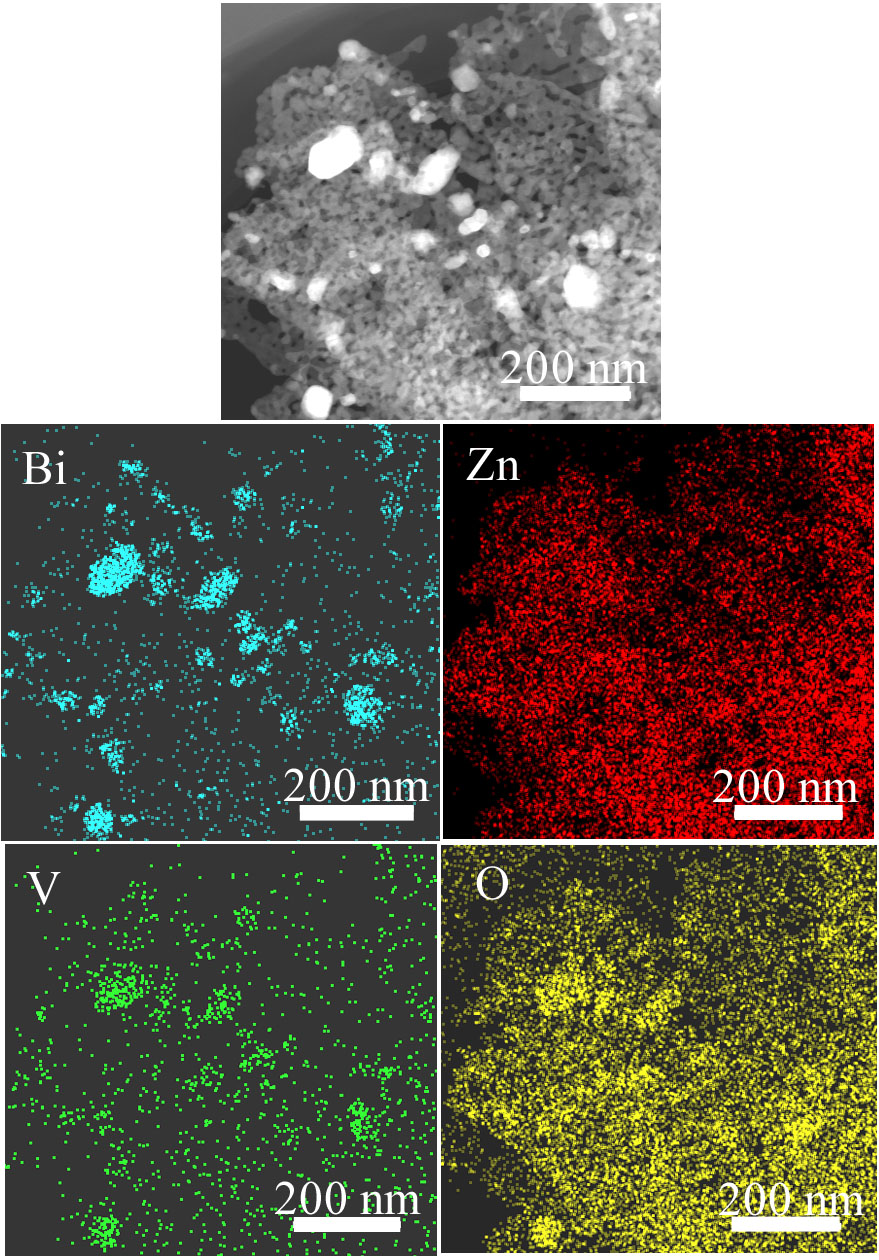


**Fig. S1** Elemental mapping of ZB_0.05, illustrating the distribution of BZVO domains and BiVO4 nanoparticles on the surface of ZnO PNSs.


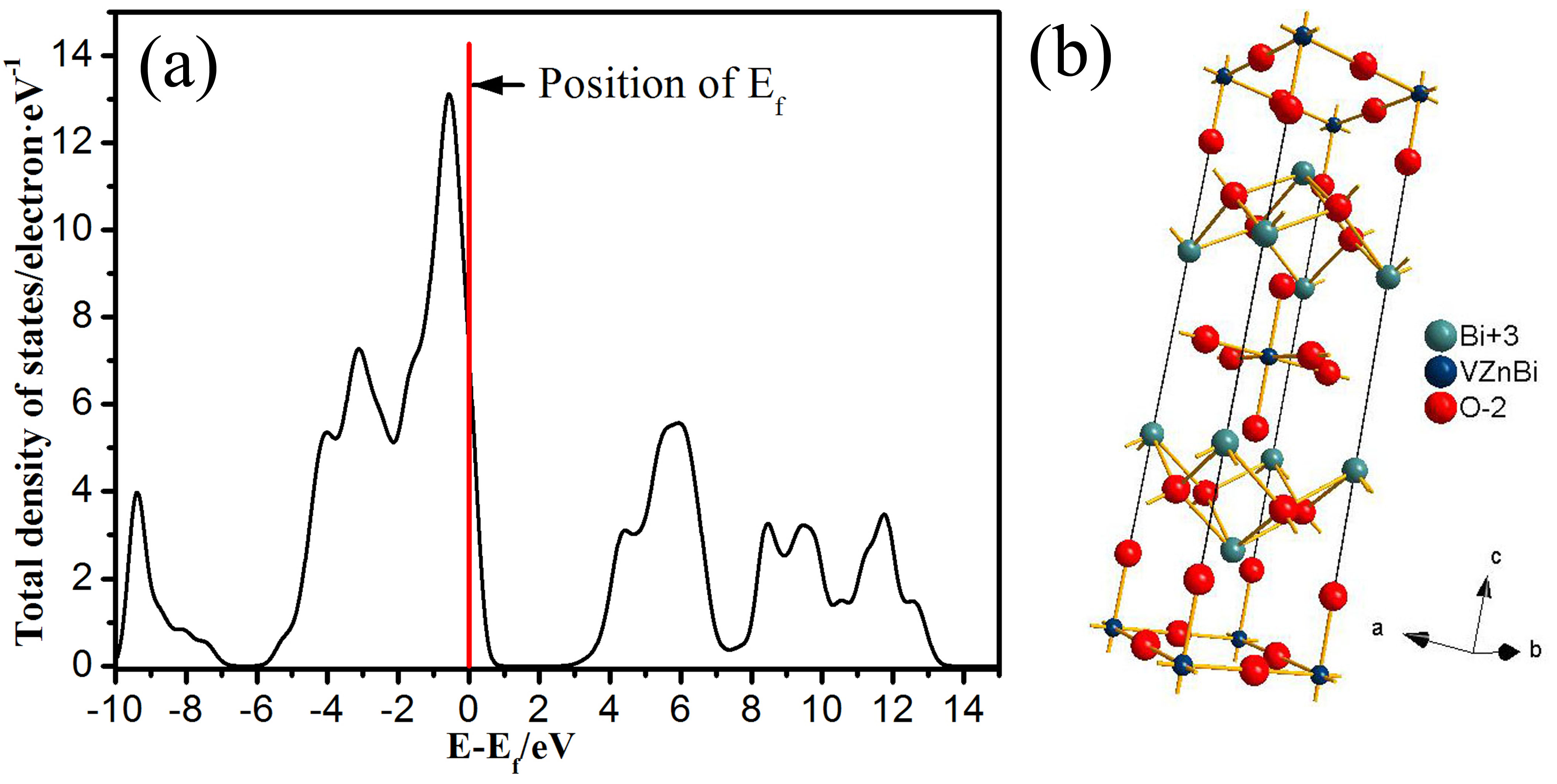


**Fig. S2** (a) DOS of BZVO solution. (b) Bulk structure for the tetragonal BZVO solution.


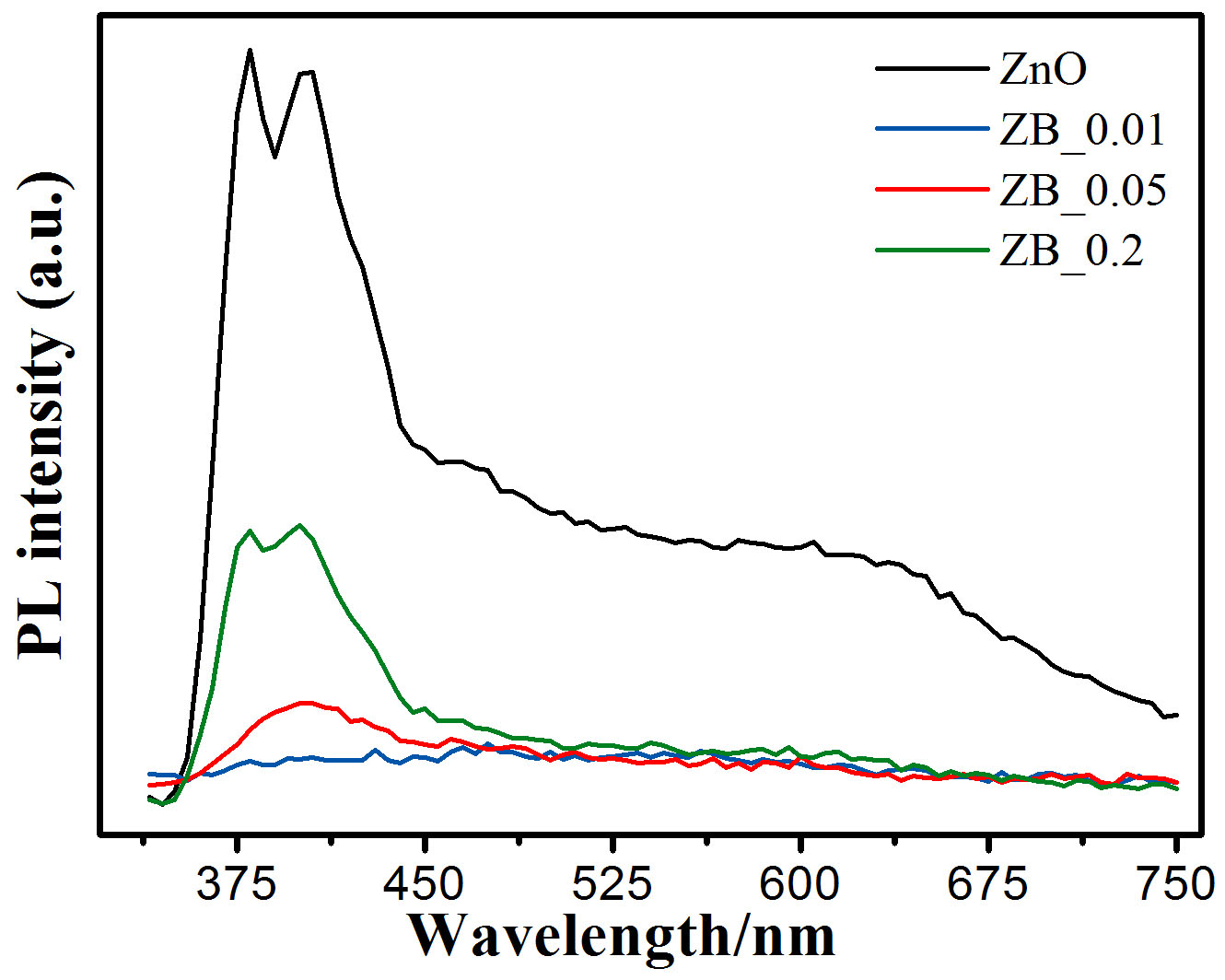


**Fig. S3** PL spectra of ZnO, ZB_0.01, ZB_0.05 and ZB_0.2 excited by the light with the wavelength of 330 nm.


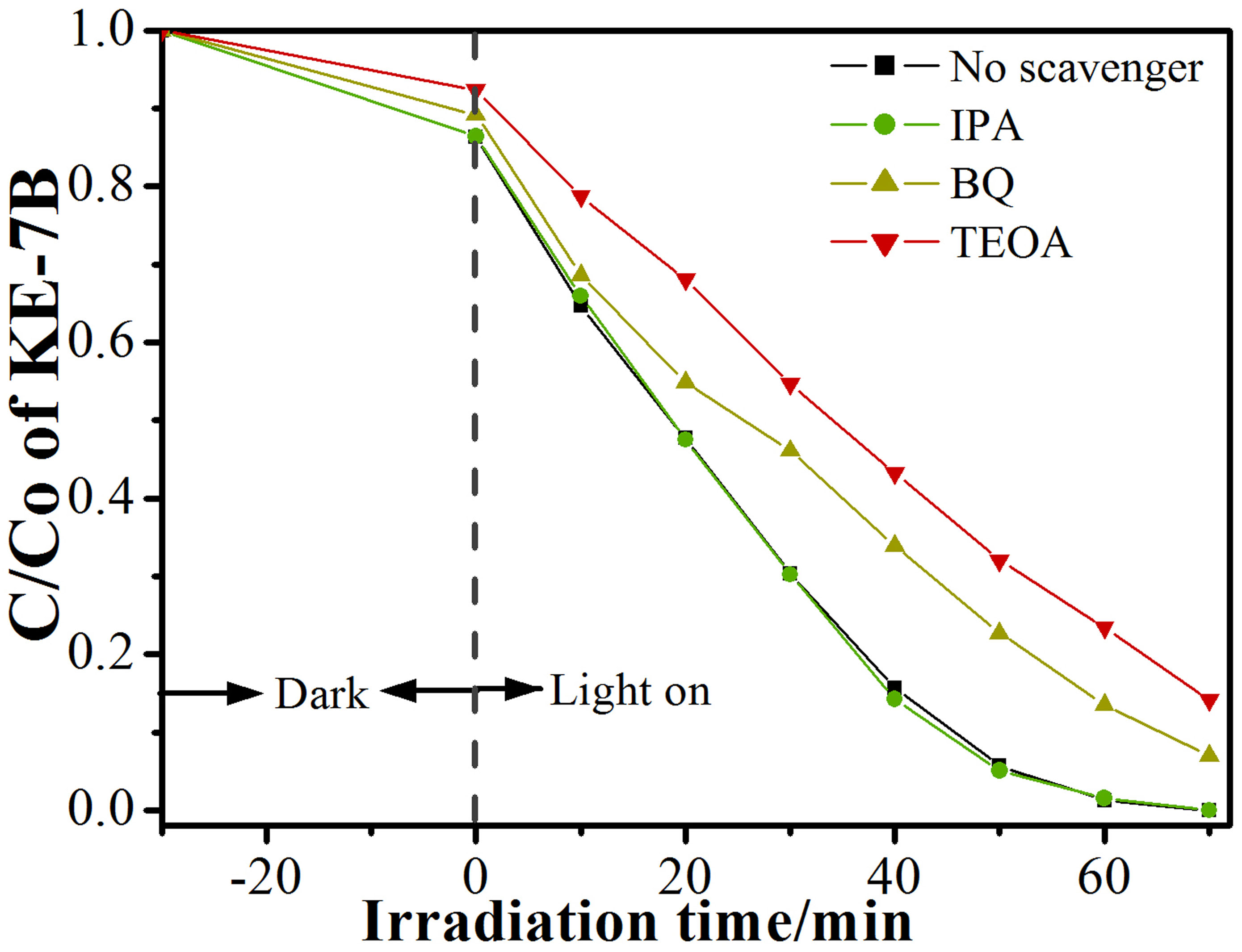


**Fig. S4** Effects of scavengers on photocatalytic degradation of KE-7B in the presence of ZB_0.01 irradiated by solar light.


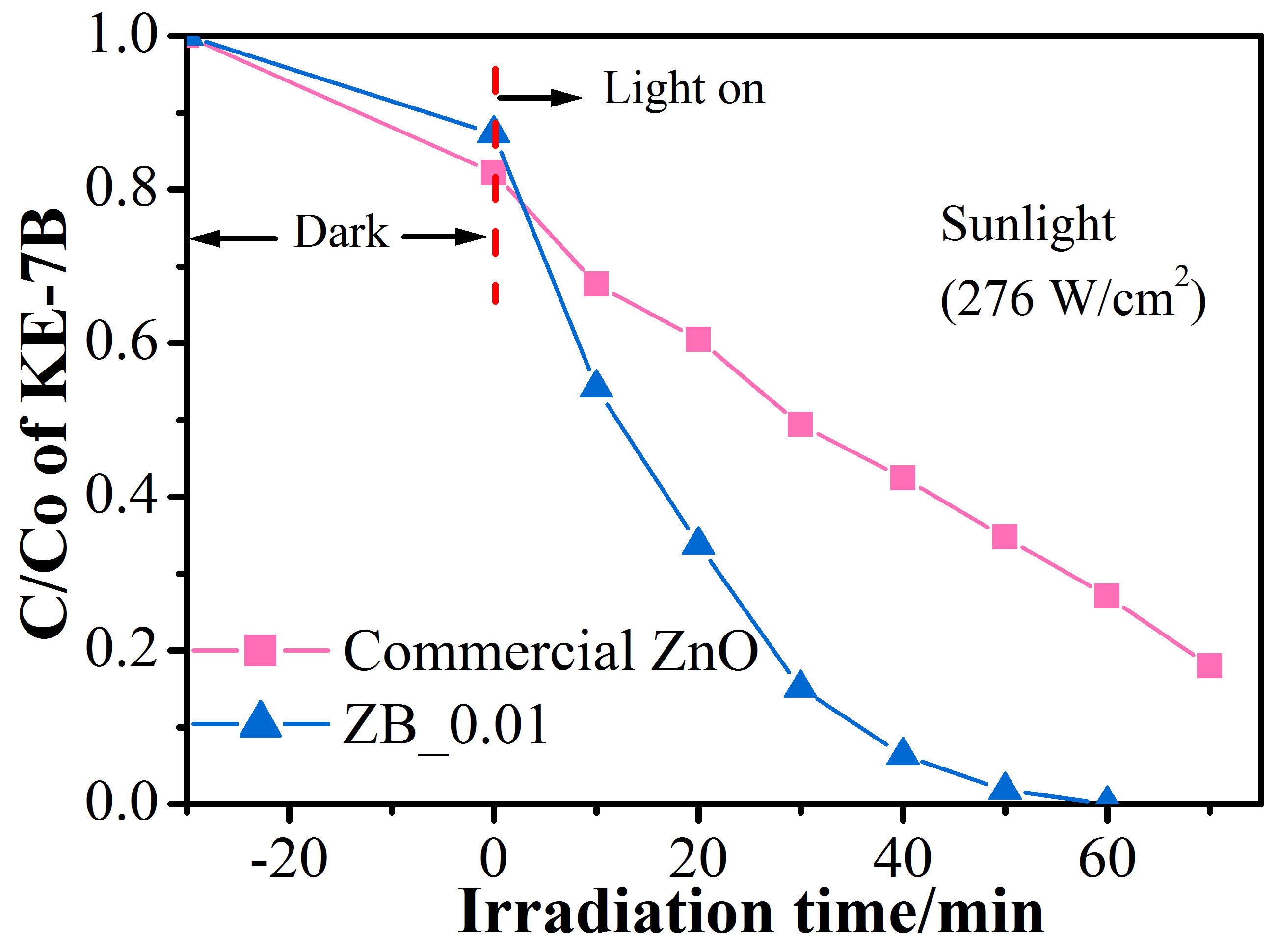


**Fig. S5** The adsorption and photocatalytic degradation on ZB_0.01 and commercial ZnO for KE-7B under sunlight illumination, indicating that ZB_0.01 performing enhanced photocatalytic activity compared to commercial ZnO.

##### **References**

##### [1] a) Kresse G, Furthmüller J (1996) Efficiency of *ab*-initio total energy calculations for metals and semiconductors using a plane-wave basis set. Comput Mater Sci 6:15–50; b) Kresse G, Furthmüller J (1996) Efficient iterative schemes for *ab* *initio* total-energy calculations using a plane-wave basis set. Phys Rev B 54:11169–11186

##### [2] a) Blöchl PE (1994) Projector augmented-wave method. Phys Rev B 50:17953–17979; b) Kresse G, Joubert D, From ultrasoft pseudopotentials to the projector augmented-wave method, Phys Rev B 59:1758–1775

[3] Perdew JP, Burke K, Ernzerhof M (1997) Generalized gradient approximation made simple, Phys Rev Lett 78:1396

[4] Kim TW, Ping Y, Galli GA, Choi KS (2015) Simultaneous enhancements in photon absorption and charge transport of bismuth vanadate photoanodes for solar water splitting, Nat Commun| 6:8769
